# Potential Effects of Geraniol on Cancer and Inflammation-Related Diseases: A Review of the Recent Research Findings

**DOI:** 10.3390/molecules28093669

**Published:** 2023-04-23

**Authors:** Rebai Ben Ammar

**Affiliations:** 1 Department of Biological Sciences, College of Science, King Faisal University, Al-Ahsa 31982, Saudi Arabia; rbenammar@kfu.edu.sa; Tel.: +966-0135899429; 2 Laboratory of Aromatic and Medicinal Plants, Center of Biotechnology of Borj-Cedria, Technopole of Borj-Cedria, P.O. Box 901, Hammam-Lif 2050, Tunisia

**Keywords:** geraniol, antioxidant, inflammation diseases, anticancer, chemo preventive effects, molecular targets

## Abstract

Geraniol (GNL), a natural monoterpene, is found in many essential oils of fruits, vegetables, and herbs, including lavender, citronella, lemongrass, and other medicinal and aromatic plants. GNL is commonly used by the cosmetic and food industries and has shown a wide spectrum of pharmacological activities including anti-inflammatory, anticancer, antimicrobial, antioxidant, and neuroprotective activities. It represents a potential anti-inflammatory agent and a promising cancer chemopreventive agent, as it has been found to be effective against a broad range of cancers, including colon, prostate, breast, lung, skin, kidney, liver, and pancreatic cancer. Moreover, GNL scavenges free radicals and preserves the activity of antioxidant enzymes. In addition, GNL induces apoptosis and cell cycle arrest, modulates multiple molecular targets, including p53 and STAT3, activates caspases, and modulates inflammation via transcriptional regulation. In the present study, different modes of action are described for GNL’s activity against cancer and inflammatory diseases. This compound protects various antioxidant enzymes, such as catalase, glutathione-S-transferase, and glutathione peroxidase. Experiments using allergic encephalomyelitis, diabetes, asthma, and carcinogenesis models showed that GNL treatment had beneficial effects with low toxicity. GNL has been shown to be effective in animal models and tumor cell lines, but there have not been any clinical studies carried out for it. The aim of the present review is to provide updated data on the potential effects of GNL on cancer and inflammation, and to enhance our understanding of molecular targets, involved pathways, and the possible use of GNL for clinical studies and therapeutic purposes in the treatment of cancer and inflammation-related diseases.

## 1. Introduction

**Figure 1:**
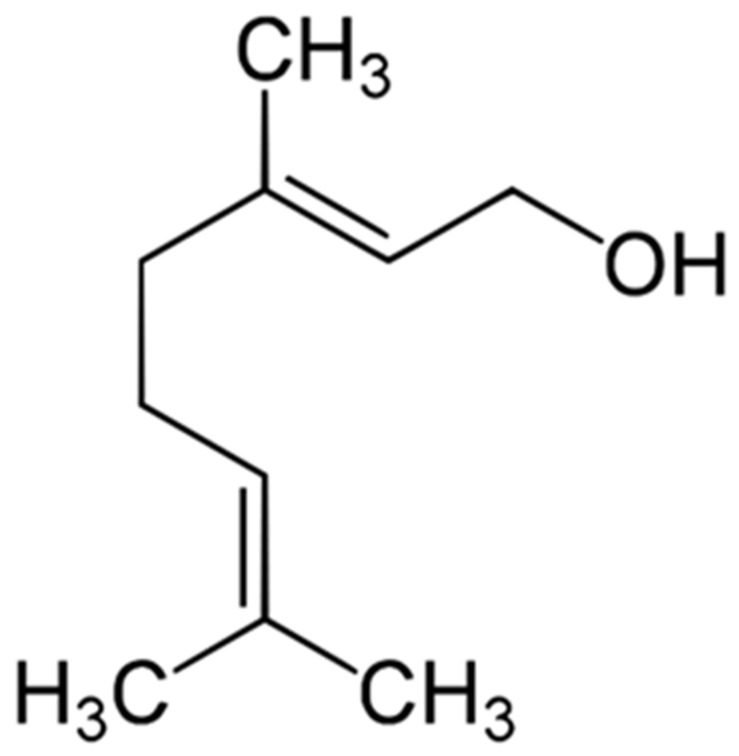
Chemical structure of Geraniol ((2E)-3,7-dimethylocta-2,6-dien-1-ol).

Recently, molecules from natural products are gaining acceptance as potentially promising complementary and alternative medicines for the treatment of various diseases [1,2,3,4,5,6,7]. Generally, pure compounds could target proteins, DNA, mRNA, and even microRNA. Studies suggest that monoterpenes might offer new chemotherapy strategies for cancer and inflammatory diseases [8,9,10,11], and accumulating evidence in the last decades has indicated that GNL is a pure botanical compound without adverse effects, exerting diverse pharmacological activities by mainly regulating protein expression. The acyclic monoterpene geraniol ((2E)-3,7-dimethylocta-2,6-dien-1-ol) is frequently found in the essential oils of many plant species (Figure 1). Lemongrass, rose, lavender, and other aromatic plants have high concentrations of Geraniol, which was shown to possess various pharmacological properties, including antioxidant [12], anti-inflammatory [13], antimicrobial [14], and antitumor activities [15], through multiple signaling pathway regulation in diverse biological processes [16,17,18]. Moreover, many studies have found that GNL inhibits cancer cell growth in vitro via the signal transduction pathway, leading to apoptosis [19,20,21,22]. Geraniol exerts in vitro and in vivo antitumor activity against murine leukemia, hepatoma, and melanoma cells [23,24]. Furthermore, the biochemical, molecular, and histological studies conducted so far indicate that GNL has antioxidant and anti-inflammatory properties. Thus, it is believed that this compound, having a strong preventive potential, can protect against oxidative and inflammatory changes [25]. Further investigation demonstrated that GNL promotes the metabolism of inflammatory cells, increases GSH content, and stimulates antioxidant enzyme activities [1]. Ji et al. [26] investigated GNL’s immunosuppressive properties by using in vitro lymphocyte proliferation assays and an in vivo rat cardiac allograft transplant model, revealing that GNL can prevent acute allograft rejection. Although many publications have been published about its anticancer activity, the mechanism of GNL molecular activity has still not been fully explained. This review aims to extend previous review findings [1,18,19,25,27] by providing an updated overview of the literature on the potential effects of GNL on cancer and inflammation-related diseases, specifically focusing on experimental study findings which enhance our understanding of molecular targets and pathways involved in antioxidant/anti-inflammatory and anticancer effects of GNL, and the possible use of this natural monoterpene for the treatment of cancer and various inflammation related-diseases. Further, the present study aims to provide a mechanistic insight into GNL’s potential as an anti-inflammatory and anticancer agent, paving the way for future experimental and clinical studies, as well as for therapeutic purposes.

## 2. Research Methodology

To gather updated data on the potential effects of GNL on cancer and inflammation-related diseases, we used the most well-known scientific search engines: PubMed, Web of Science, ScienceDirect, Wiley Online, Google Scholar, SpringerLink, and Scopus. Data were mainly collected from several sources including journal articles, books, book chapters, and scientific reports. The search for this information was carried out between November 2022 to March 2023. The scientific literature published in the years 2020 to 2023 was searched. However, several papers published before 2020 were also included for insight in the introduction and explanation. We used Geraniol either alone or combined with ‘anti-inflammatory’, ‘antitumor’, ‘anticancer’, ‘antiproliferative’, ‘cytotoxicity’, and ‘antioxidant’ as the key words for the literature searches. The internet search generated 1147 articles in total. Duplicate articles and articles with similar results were excluded. Publications in other languages than English and those with limited raw data were also excluded. Only 104 articles were finally included in this study after reading the titles, abstracts, and whole papers.

## 3. Anti-Inflammatory and Antioxidant Properties of GNL

**Figure 2:**
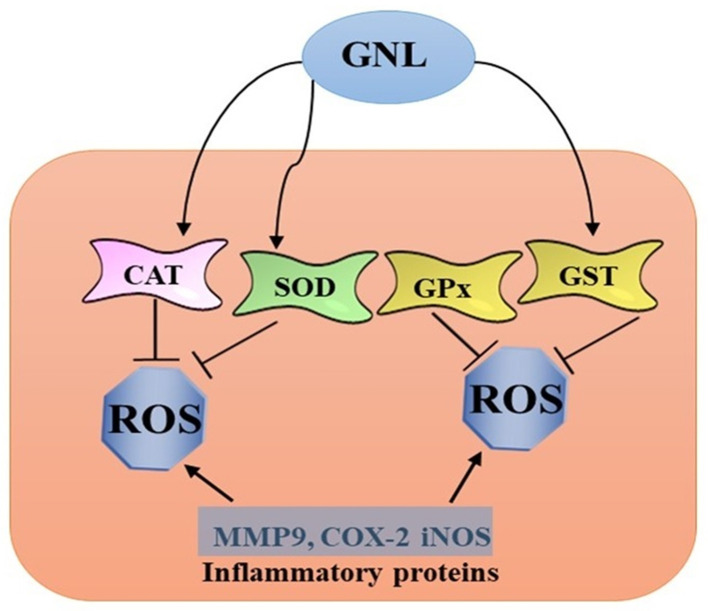
GNL enhances antioxidant enzymes and inhibits inflammatory proteins.

The immune system reacts non-specifically to injury or infection when inflammation occurs [25,28,29]. Research has revealed that chronic inflammation can cause diseases such as cancer, cardiovascular disease, and neurological disorders [30,31,32,33,34]. Since GNL has anti-inflammatory and antioxidant effects, scientists are studying its molecular mechanisms and potential applications in treating inflammation. El Azab and Abdulmalek [35] evaluated the ameliorative effect of GNL on age-related multiple inflammation and neuronal impairments in rats fed with high-fat diet (HFD). Their results revealed a drop in proinflammatory cytokines (TNF-α and IL-6) and leptin while boosting adiponectin in GNL-supplemented rats. The liver, kidney, and lipid profiles were improved in GNL-HFD-treated groups. In addition, GNL suppressed acetylcholinesterase (AChE) activity and alleviated oxidative stress by boosting neuronal reduced glutathione (GSH), catalase (CAT), glutathione-S-transferase (GST), and superoxide dismutase (SOD) activities. It lowered malondialdehyde concentration (TBARS), nitric oxide (NO), and xanthine oxidase (XO), and restored the structural damage to the brain tissue caused by HFD. Compared with model rats, GNL boosted learning and memory function and ameliorated the inflammation status in the brain by lowering the protein levels of IL-1β, iNOS, NF-κBp65, and COX-2 (Figure 2).

Furthermore, the supplementation of GNL reversed the oxidative and inflammation changes associated with aging. According to El Azab and Abdulmalek [35], dietary GNL supplementation was effective in modifying age-related neuroinflammation and oxidative stress in rats, suggesting the use of GNL as a noninvasive natural compound for controlling age- and diet-associated neuronal impairments and toxicity. In the same framework, AlAsmari et al. [36] investigated the potential effect of GNL on the modulation of doxorubicin-induced kidney damage in rats. A single doxorubicin injection triggered kidney impairment, as evidenced by the altered blood urea nitrogen, serum creatinine, and albumin parameters; it also caused kidney histological changes. Furthermore, doxorubicin increased lipid peroxidation while lowering catalase activity, reduced glutathione, and the expression of glutathione peroxidase and superoxide dismutase. Moreover, prophylactic treatment with GNL preserved most kidney histological characteristics in a dose-dependent manner, showing that GNL could protect against doxorubicin-mediated kidney dysfunction [36].

## 4. Anti-Free Radical Properties of GNL

GNL decreases the brain damage induced by ischemia/reperfusion in mice [29]. In RAW264.7 rat macrophages, GNL suppressed reactive oxygen species (ROS) levels induced by lipopolysaccharides [37]. COX-2 protein levels and mRNA levels were significantly attenuated, but the cytosolic degradation of IBα and upregulation of NF-κB p65 in the nucleus were reversed. LPS/D-GalN-induced FHF was inhibited by GNL through the inhibition of inflammation and an increase in PPARγ expression [38]. Another experiment showed the aforementioned inhibitory effects were mediated by the inhibition of iNOS and COX2 enzyme induction. The induction of iNOS and COX-2 has also been reported in cyclophosphamide-induced cystitis in rats [39]. In addition, iNOS and COX-2 inhibitors have been shown to reduce hyperactivity. Mohamed et al. inspected the GNL’s reno-protective effects against renal I/R damage with further analysis of embedded mechanisms of action through scrutinizing the Nrf-2/HO-1/NQO-1 and TLR2,4/MYD88/NFκB signaling pathways [40]. Their results showed that Renal I/R rats experienced severely compromised renal functions, histological alteration, oxidative stress status, escalated Nrf-2/HO-1/NQO-1, and amplified TLR2,4/MYD88/NFκB. GNL administration ameliorated renal function, alleviated histological changes, and enhanced Nrf-2/HO-1/NQO-1 with a subsequent intensification of antioxidant enzyme activities. GNL declined TLR2,4/MYD88/NFκB with subsequent TNF-α, IFN-γ, MCP-1 drop, Bax, caspase-3, and caspase-9 reduction IL-10 and Bcl-2 augmentation. GNL, therefore, might protect against renal I/R via the inhibition of the TLR2,4/MYD88/NFκB pathway, mediating anti-inflammation and activation of the Nrf2 pathway, this intervening in antioxidative activities [40]. In general, GNL has been shown to enhance the effect of antioxidant enzymes and inhibit inflammatory proteins.

## 5. Effects of GNL on Autoimmune Diseases

Inflammation triggers the immune system to react non-specifically against injury or infection. Chronic or continuous inflammatory diseases can result from uncontrolled inflammation [29]. Studies have shown that GNL can help with diabetes, arthritis, asthma, and other autoimmune diseases [41,42,43]. In a streptozotocin diabetic mouse model, GNL abrogated hyperglycemic and hypoinsulinemic responses [44]. Ischemia/reperfusion-induced brain damage was shown to be decreased by GNL in mice [45]; in this study, GNL suppressed TNF-α, iNOS, and COX-2 levels. Furthermore, GNL, Bax, and caspase-3 and -9 reduced antiapoptotic activity in liver tissue. The Nrf2/HO-1 antioxidant pathway is activated by GNL, making it a promising hepatoprotective agent. In streptozotocin or streptozotocin–nicotinamide diabetic rat models, GNL reversed both elevated glucose and low insulin levels [42,46]. GNL may have therapeutic effects in the treatment of diabetes by modulating serum glucose and insulin levels. Streptozotocin diabetic rats showed improvement in neuropathy and nephropathy with administration of GNL, suggesting that it could help to prevent diabetes-related diseases [47,48]. Additionally, this monoterpene reduced collagen-induced arthritis in mice [49]. In another study, GNL reduced serum nitric oxide, urea, and creatinine levels, as well as prevented kidney dysfunction, which is common in nephrotoxic animal models [50]. GNL has also been shown to decrease alkaline phosphatase expression [51]. A nonreceptor tyrosine kinase called Tyk2 was inhibited by GNL in mice induced by OVA [40]. Through modulating Tyk2-STAT1/3, GNL alleviates airway inflammation. LPS was also shown to stimulate STAT1/3 signaling while downregulating Tyk-inhibiting SOCS3 expression [52].

## 6. Glutathione Modulation by GNL

In a study by Stobiecka et al. [52], GNL was found to be a powerful scavenger of free radicals [52]. These findings are in line with [53], in which it was shown that GNL inhibits iron-dependent microsomal lipid peroxidation. These results suggest that GNL might have a role in oxidative stress prevention and treatment. Various sources of free radicals or superoxide radicals can be neutralized by antioxidant enzymes. In CCl4-induced oxidative stress rats, Mostafa, et al. [54] showed that GNL reversed the expression of catalase, glutathione peroxidase, and glutathione-S-transferase, and reduced glutathione in the liver tissues [54]. GNL also reverses isoproterenol’s suppression of catalase, glutathione peroxidase and glutathione-S-transferase activity, and mRNA expression [55]. Thus, GNL might be useful for reducing the adverse effects of inflammatory disorders caused by free radicals. Glutathione is a tripeptide used in drug detoxification that can prevent ROS damage [20,56]. Pronin et al. [57] reported that GNL improved experimental anti-encephalitis in vitro [57]. In addition, Kandeil et al. [58] noted that GNL reversed the reduced levels of glutathione, glutathione peroxidase, and catalase levels in rats treated with cisplatin [58]. GNL’s effects on glutathione levels in the body need to be investigated more closely, since such findings might explain its role in inflammation suppression or cancer prevention.

## 7. Transcriptional Effects of GNL on Inflammation

**Figure 3:**
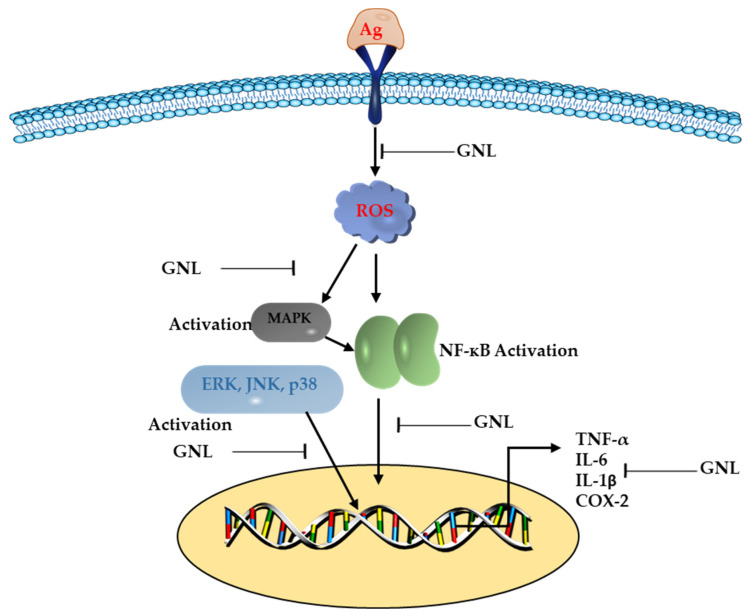
GNL inhibits inflammation by blocking NF-ĸB.

A wide range of stimuli, including stress, bacteria, viruses, cytokines, and free radicals, can trigger NF-ĸB. This transcription factor regulates the expression of a variety of genes, including enzymes, cytokines, and cell-cycle regulators [58,59,60]. In LPS-induced acute lung injury, Jiang et al. [61] reported that GNL inhibited the production of pro-inflammatory cytokines in BALB, including TNF-α, IL-1β, and IL-6, as well as the MAPKs and NF-kB signaling pathways [61]. Another study showed that monoterpene fractions suppress TNF-α-induced NF-kB activation [13]. A previous study suggested that GNL inhibited NF-kB translocation into the nucleus in Ox-LDL-stimulated inflammation in HUVECs [62]. In addition, Lee et al. [63] found that an extract of lemongrass abrogates LPS-induced NF-kB signaling in RAW264.7 cells by targeting IKKb [63]. Several inflammatory disease models have been studied and the benefits of modulating NF-kB with GNL have been demonstrated. Treatment with GNL had previously been shown to prevent or ameliorate Japanese encephalomyelitis, possibly by inhibiting NF-kB [64]. According to a recent study [27,43], GNL may protect against rheumatoid arthritis through inhibition of NFkB-p65, p38, and ERK1/2 phosphorylation (Figure 3). NF-kB suppression by GNL probably plays a significant role in its anti-inflammatory properties. Angiogenesis, metastasis, differentiation, proliferation, and apoptosis are all controlled by STAT3. Many carcinomas are constitutively activated by STAT3, and it interferes with tumor growth at different levels [65,66,67]. A reduction in survivin levels caused by GNL-induced apoptosis was shown by Kuzu et al. [68]. MG132 inhibits proteasomal degradation in GNL-treated cells, restoring survivin levels [69]. In other words, survivin attenuates GNL-induced apoptosis. GNL reduces survivin protein levels by downregulating phosphorylated STAT3. GNL also inhibits survivin by blocking serine/threonine kinase activity because active phosphorylated STAT3 increases survivin stability. GNL inhibits the inhibitors of apoptosis, such as Bclxl, Mcl and Bcl-2, which belong to the anti-apoptotic Bcl-2 family [70]. GNL also reduces IL-8 production by LPS-stimulated human pulmonary epithelial cells [71]. As well as its antioxidant properties, GNL has PARP-1-inhibiting activity [72].

## 8. Anticancer and Chemo Preventive Effects of GNL

Chemoprevention has emerged as a new strategy for fighting cancer, preventing and reducing cancer risk via the ingestion or administration of natural or synthetic chemicals. In the search for new cancer chemopreventive agents, many plant constituents have been evaluated for their chemopreventive activities against cancers over the past few years [73]. The possible mechanism of GNL in relation to antioxidant status is the inhibition of free radical formation and reduced cancer incidence. It was found that GNL treatment is effective during the post-initiation phase of carcinogenesis. An increase in the activity of antioxidants and a decrease in the level of marker enzymes suggest that GNL reduces the adverse effects of cancer. Furthermore, a reduction in the levels of glycoprotein components during treatment with GNL indicates that acyclic monoterpene alcohol has the ability to suppress malignancy by modulating cell transformation through controlling cell proliferation. GNL plays a very important cytoprotective role against B(a)P-induced lung carcinogenesis [74].

In many countries, cancer is one of the most common diseases, with an increasing incidence rate every year [75]. There is an urgent need for a cure to this disease, as treatment is expensive, and complications frequently lead to death. GNL has been shown to inhibit the in vivo growth of cancer in mice with benzo(a)pyrene-induced lung cancer [73]. The antioxidant properties of GNL have been linked to the prevention of chemical-induced cancer. Scientists are researching the molecular mechanism(s) involved and are evaluating GNL’s significance in treating cancer on the basis of its potential. A number of in vitro and in vivo studies have shown that GNL inhibits tumorigenesis and prevents carcinogenesis. Elsayad and Adeshina evaluated the antitumor effects of GNL on oral cancer [33]. They have shown that GNL treatment significantly suppressed oral squamous cell carcinoma (OSCC) cell proliferation and migration in vitro and tumor growth in vivo in a time- and dose-dependent manner. In addition, they have shown that GNL treatment significantly caused OSCC apoptosis and blocked Phosphatidylinositol-3-kinase/protein kinase B (PI3K/AKT) signaling activation concurrently. They also found that GNL administration did not affect the body weight on tumor-bearing mice, confirming the GNL safety and showing that this compound may serve as a promising anticancer drug for oral cancer treatment [33].

## 9. Effect of GNL on Cell Proliferation

Hyper-proliferative cancerous cells are well-established targets for cancer treatment [75]. GNL helps to identify transcription factors controlling the gene expression of HaCaT keratinocytes [76,77]. Several kinds of carcinoma, including glioma and glioblastoma, breast carcinoma, osteosarcoma, and colorectal carcinoma, are inhibited by this compound [27,33,50,78,79,80]. 4NQO-induced oral cancer was inhibited by GNL in rats. GNL inhibits phase I enzymes and blocks the bioactivation of 4NQO to 4HAQO, which is cancer-causing, leading to a significant reduction in tumor volume and number [81]. Thymidine kinase and thymidylate synthase were effectively reduced by GNL. In Swiss nu/nu mice with implanted human TC-118 cancer cells, simultaneous administration of 5-fluorouracyl and GNL resulted in 53% tumor volume reduction, versus 26% for GNL alone and no difference for 5-fluorouracyl alone [15]. When chronically exposed to Fe-NTA, rats treated with rat GNL showed a significant decrease in p53 protein levels and a significant increase in caspase-3, -8, and -9, some of which are classic markers of inflammation, proliferation, and apoptosis [82]. In hamsters treated with DMBA, GNL completely prevented the formation of tumors in the cheek mucosa, affected the expression of p53 and Bcl-2, and increased the levels of Bax protein and caspase-3 and -9. GNL prevents cell proliferation markers, inflammation, apoptosis, and angiogenesis at tumor sites after oral administration [67]. Duan et al. [83] studied the potential effect of a multi-bioresponsive self-assembled nano drug delivery system based on hyaluronic acid and GNL against liver cancer [83]. A multi-bioresponsive self-assembled nano-drug delivery system (HSSG) was constructed by conjugating GNL to hyaluronic acid (HA) via a disulfide bond. According to Duan et al. [68], results of fluorescence microscopy and flow cytometry showed that HSSG NPs were uptaken by hepatocellular carcinoma cell lines HepG2 and Huh7 via CD44 receptor-mediated internalization. Studies on H22 tumor-bearing mice revealed that HSSG NPs could effectively accumulate at the tumor site for a long period. In vitro and in vivo studies showed that HSSG NPs significantly promoted the death of cancer cells while reducing the toxicity. Therefore, the HSSG NPs have great potential in the treatment of tumors [83]. Based on these results, GNL may be helpful in treating different types of malignancy, while having limited effects on normal cells.

## 10. Cancer Cells Respond to GNL in A Pro-Apoptotic Way

**Figure 4:**
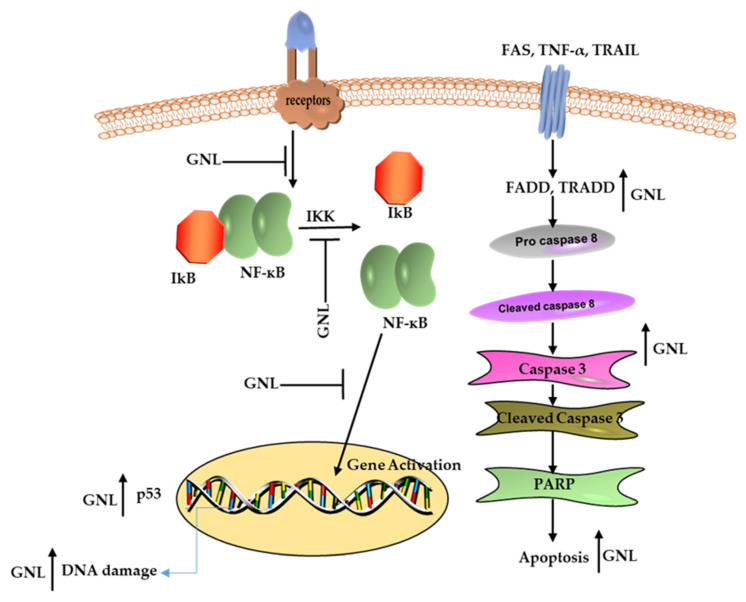
Mechanisms of action of GNL in cancer treatment.

The ability of chemotherapeutic drugs to induce apoptosis determines their effectiveness in cancer treatment. As a cancer preventative or therapeutic agent, GNL has been shown to have beneficial effects on various types of cancer in humans but does not affect normal physiology by regulating the cell cycle and/or inducing apoptosis. According to Carnesecchi et al. [84], GNL induced apoptosis and growth inhibition in the human HCT116 colon cancer cell line. GNL activated caspase-3 cleavage and released cytochrome c from mitochondria. Bcl-2 family proteins such as PUMA play a key role in this [19,85]. In HCT116 cells, GNL also phosphorylated ATM and H2AX, and inhibited ATM with a chemical inhibitor that abrogated the downstream apoptotic cascades [77]. GNL treatment decreased Bcl-xL protein levels in ovarian cancer cells, while Bax, p53, and Bad protein levels were upregulated [70,86]. GNL also induced more apoptosis than either agent used alone in human colon cancer SW480 cells [87]. By regulating pro-apoptotic and anti-apoptotic proteins in the intrinsic apoptosis pathway, GNL induces apoptosis in ovarian cancer cells. Following GNL treatment, Bclxl protein levels were decreased in ovarian cancer cells, while p53, Bad, and Bax protein levels were upregulated (Figure 4). In addition, Akt was inactivated, the mitochondrial phase of apoptosis was activated, cytochrome c was released, caspase 3 was activated, and cells death eventuated [33]. Duncan et al. [88] observed that GNL inhibits cyclin-dependent kinase 2, which regulates cell proliferation, anti-apoptotic gene products, and metastatic transcription factors [88]. According to Shoff et al. [12], GNL inhibits liver HMG-CoA, which participates in cholesterol biosynthesis in mammals, as well as the cell cycle. As a result, its inhibitors can effectively demonstrate an anticancer activity by stopping the cell cycle between the G1 and S phases [12]. Zhuang et al. [89] evaluated the GNL protective effect on Helicobacter pylori-induced human gastric cancer signaling by increasing peroxiredoxin-1 expression in human gastric epithelial cells (GES-1) [89]. Indeed, GNL inhibits *H. pylori*-induced gastric carcinogen signaling by preventing ROS formation, cytotoxicity, and apoptosis in GES-1 cells. In addition, GNL prevents *H. pylori*-induced antioxidant depletion induced by nuclear fragmentation, damage of reactive DNA, and malondialdehyde. Furthermore, GNL potentially reduced the expression of phosphorylated mitogen and activated protein kinases (MAPKs) proteins such as p38 MAPK, tumor necrosis factor-alpha (TNF-α), c-Jun N-terminal kinase (c-JNK), extracellular signal-regulated kinase-1 (ERK1), interleukin-6 (IL-6), and cyclooxygenase-2 (COX-2) in GES-1 infected with *H. pylori*. Thus, GNL protects against *H. pylori*-concomitant infection, and its resistance may be a possible method of preventing gastric cancer caused by *H. pylori* [89].

The antitumoral activities of GNL against different types of cancer and using different modes of study are summarized in Table 1.

## 11. Effect of GNL on Metastasis and Angiogenesis Inhibition

In numerous studies, GNL has been reported to inhibit cancer metastasis and angiogenesis. Researchers have found that monoterpene modulates apoptosis, angiogenesis, inflammation, and metastasis signaling pathways. GNL seems to inhibit cancer cell growth and angiogenesis and induce cancer cell apoptosis, while preserving normal cell viability, and in some cases even protecting it. Part of the purpose of this review was to summarize information about GNL and provide insight into its potential chemo-preventive effects. VEGF gene expression at both the mRNA and protein levels is significantly reduced in endothelial-like eEND2 cells by GNL [103]. GNL inhibited tumor growth in 7,12-dimethylbenz[a]anthracene (DMBA)-induced hamster mice [83]. El-Ella [104] found that in A549 lung cancer cells treated with GNL, downregulation of HIF-1alpha, a VEGF regulator, occurred [104]. With onset of hypoxia, GNL downregulates NF-ĸB and VEGF expression levels, resulting in the downregulation of inflammatory and angiogenic markers. GNL also decreases autophagy through downregulation of BNIP3 and beclin-1 expression, which increases apoptotic cell death through HIF-1α signaling.

## 12. An Overview of GNL Bioavailability

Using NCM460 cells derived from primary human colon mucosa cells, tests were performed to simulate GNL penetration through the in vitro intestinal barrier. Additionally, GNL can potentially be transported from the intestinal lumen into the bloodstream without any degradation in the digestive tract, which indicates high penetration through the monolayers of cells. Plant fibers absorbed geraniol at 16% bioavailability, so it can reach the colon through the intestines [25,102]. Even at concentrations as high as 300 g/mL, geraniol is excreted from the bloodstream within about 12 min after intravenous administration. Based on its binding to blood proteins and cellular components, and its penetration into lipid compartments, researchers have suggested that in this case, a high concentration of GNL could damage mitochondrial depletion, leading to apoptosis. In studies performed using Sprague Dawley rats, the absolute availability of GNL was 92% with administration of GNL emulsified in glycerol. The maximum blood concentration was found after 30 min and was about 270 µg/mL. It is worth emphasizing that this was similar to the value obtained after intravenous administration of the same dose of GNL [16]. The concentration of GNL in the cerebrospinal fluid of the rats dropped rapidly over time, similarly to the blood concentration [16].

## 13. Conclusions and Future Prospects

Different modes of action have been shown to occur with GNL application against cancer and inflammation. Various antioxidant enzymes, such as catalase, glutathione peroxidase, and glutathione-S-transferase, are protected by this compound, which scavenges free radicals and superoxide radicals. Experiments using allergic encephalomyelitis, diabetes, asthma, and carcinogenesis models have shown that GNL treatment has beneficial effects. There are several different anticancer mechanisms, including the inhibition of proliferation, the induction of cell cycle arrest, the induction of apoptosis, synergy with conventional medicines, and ROS generation. Additionally, GNL can attenuate the toxicity associated with conventional medicines without compromising their effectiveness. GNL has been shown to target a variety of molecules in cancer cell lines; however, animal models are needed to obtain more conclusive evidence of the molecular basis of its action. In addition, novel GNL analogs have been synthesized and found to have better anticancer, anti-inflammatory and antioxidant properties. It has been shown to be effective in animal models and tumor cell lines, but there have not yet been any clinical studies carried out to further assess these effects. More studies are needed before GNL can be developed into a drug for cancer treatment and the treatment of inflammatory diseases. For GNL to be commercialized as a cancer preventative and therapeutic agent, substantial evidence from epidemiological research and clinical trials will be required. Further, the present study provides a mechanistic insight into GNL’s potential as an anti-inflammatory and anticancer agent, paving the way for future experimental studies. Overall, these findings show that GNL modulates molecular targets and pathways to trigger apoptosis in cancer cells. GNL is rapidly moving into clinical trials, so these targets could be exploited for therapeutic purposes in future.

## Figures and Tables

**Table 1:** Antitumor activities of GNL.

Type of Cancer	Mode of Study	Cell Lines/Animal Model	Target	Effect	References
Skin cancer	In vivo/In vitro	Swiss albino mice PC-3, A431, and A549 cells Swiss albino mice	Ras/Raf/ERK1/2 ornithine decarboxylase, LOX-5, and hyaluronidase Phase II and Antioxidants	Apoptosis Anti-proliferation Chemoprevention	[90,91,92]
Pancreatic cancer	In vitro	BXPC-3 cells	DNA damage	Apoptosis, Anti-proliferative	[93,94]
Prostate cancer	In vitro	PC-3	E2F8 expression	Anti-proliferative	[95]
Colon cancer	In vitro	HT-29	DNA damage	Apoptosis	[96]
Liver cancer	In vitro/In vivo	HepG2/Rats	Mevalonate pathway, HMGCR, DNA damage and ERK, NFkB	Apoptosis; anti-proliferative	[83,97,98,99,100]
Lung Cancer	In vitro/In vivo	A549, Albino mice	Mevalonate pathway, DNA damage, Chemoprevention	Apoptosis; anti-proliferative	[101,102]

## Data Availability

Not applicable.
